# Finite Element Modeling of Magnitude and Location of Brain Micromotion Induced Strain for Intracortical Implants

**DOI:** 10.3389/fnins.2021.727715

**Published:** 2022-01-06

**Authors:** Ali Al Abed, Jason Amatoury, Massoud Khraiche

**Affiliations:** ^1^Department of Mechanical Engineering, American University of Beirut, Beirut, Lebanon; ^2^Sleep and Upper Airway Research Group, Biomedical Engineering Program, American University of Beirut, Beirut, Lebanon; ^3^Neural Engineering and Nanobiosensors Group, Biomedical Engineering Program, American University of Beirut, Beirut, Lebanon

**Keywords:** intracortical, micromotion, FEM, implants, brain, neuron, strain, glia

## Abstract

Micromotion-induced stress remains one of the main determinants of life of intracortical implants. This is due to high stress leading to tissue injury, which in turn leads to an immune response coupled with a significant reduction in the nearby neural population and subsequent isolation of the implant. In this work, we develop a finite element model of the intracortical probe-tissue interface to study the effect of implant micromotion, implant thickness, and material properties on the strain levels induced in brain tissue. Our results showed that for stiff implants, the strain magnitude is dependent on the magnitude of the motion, where a micromotion increase from 1 to 10 μm induced an increase in the strain by an order of magnitude. For higher displacement over 10 μm, the change in the strain was relatively smaller. We also showed that displacement magnitude has no impact on the location of maximum strain and addressed the conflicting results in the literature. Further, we explored the effect of different probe materials [i.e., silicon, polyimide (PI), and polyvinyl acetate nanocomposite (PVAc-NC)] on the magnitude, location, and distribution of strain. Finally, we showed that strain distribution across cortical implants was in line with published results on the size of the typical glial response to the neural probe, further reaffirming that strain can be a precursor to the glial response.

## Introduction

**FIGURE 1:**
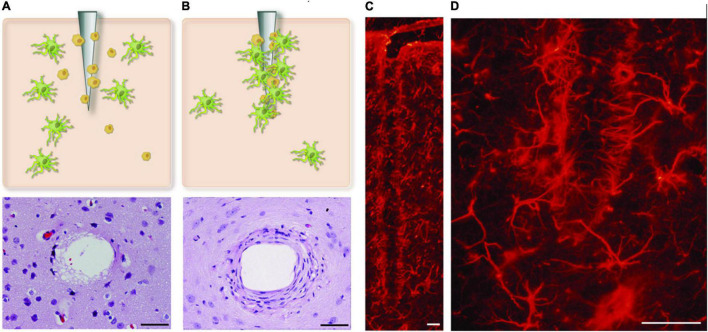
Glial encapsulation of intracortical microelectrode. **(A,B)** Shows two stages of glial activation where astrocytes and microglial cells are activated and incapsulate probe at the site of injury. **(C,D)** The reactive astrocytes, immunohistochemically labeled here for GFAP, encapsulate the neural probes forming a dense cellular sheath. Figure adapted from Marin and Fernandez (2010). Scale bar = 50 μm.

High-fidelity from intracortical microelectrodes recordings are central for the efforts to understand the complexity of neural networks in awake patients or repair/bridge damaged pathways through open or potentially closed-loop prosthetic intervention (Chou et al., 2015; Lindner et al., 2019). The Michigan silicon-based microelectrode, developed at the University of Michigan by Kensall Wise and his colleagues, was the first cellular level intracortical microelectrode (McAlpine, 2016). Currently, the technology is used clinically in deep brain stimulation, auditory brainstem neuroprostheses, cortical stimulation, and brain-machine interface (Paralikar, 2009; Khraiche et al., 2013a; Kook et al., 2016). Despite these applications, chronic brain implants suffer many challenges including signal loss, reduced signal-to-noise ratio, and unstable recordings over time (Kook et al., 2016). The implant/neural tissue interaction gives rise to a complex system from a biomechanical, chemical, and bioelectrical standpoint. One of the factors that can potentially contribute to limiting implant life for clinical applications of intracortical electrodes is the foreign body response at the implant-injury site (Campbell and Wu, 2018). This is characterized by a cascade of inflammatory events, which culminate in chronic inflammation, resulting in the failure of the implant over extended periods. At the center of this response is the brain immune response driven by native immune cells (glia). These cells act to encapsulate the electrode, electrically isolating it from the target tissue (Figure 1). The catalyst for the brain’s immune response includes initial injury during implantation, foreign body response to implant material and shape, and chronic micromotions of the implant. The latter is caused by breathing, heartbeats, and vascular pulsation, or external body motion such as rapid head movement (Muthuswamy et al., 2003). In addition to immune response, recent evidence points to a direct role of mechanical forces in neural modulation, including heightened functional state and a high neural firing rate (Marin and Fernandez, 2010; Khraiche et al., 2017; Fomenko et al., 2018).

The above challenges have driven research efforts toward a close evaluation of the biomechanics of intracortical microelectrode implants with a focus on the strain-induced on neural tissue at the injury site. However, these strains are very difficult to measure given their location deep in the brain. Accordingly, finite element models have been developed to measure induced strain fields resulting from a material mismatch between the brain tissue and the implanted probe (Lee et al., 2005; Subbaroyan et al., 2005; Zhu et al., 2011; Nguyen et al., 2014; Zhang et al., 2016; Mahajan et al., 2020). Past models have studied various parameters of the probe and its relationship with the strain levels and distribution. However, contradicting conclusions regarding the location of the maximum strain for various materials and range of micromotions are present (REFS). In this work, we focus on investigating the impact of the range of micromotion of the brain (vascular and respiratory induced motion) on the maximum strain for a stiff silicon-based microelectrode. We also compare strain across several candidate materials for brain implants including stiff silicon, polyimide, soft hypothetical materials, and polyvinyl acetate nanocomposite (PVAc-NC). PVAc-NC is a stimuli-responsive polymer nanocomposite that changes from rigid to soft following insertion into the brain, making it more mechanically compliant with the brain tissue (Nguyen et al., 2014). We also investigate the surrounding strain distribution for stiff silicon versus PVAc-NC compliant materials across the length and surrounding region of the probe and compare it to past histological evaluation of the injury site. Finally, we investigate two different probe thicknesses of a compliant implant on the maximum strain.

## Materials and Methods

We modeled an intracortical microelectrode placed in the brain. The displacement of the brain due to respiration ranges between 2 and 25 μm and smaller displacements due to vascular pulsations in the brain can range between 1 and 4 μm (Muthuswamy et al., 2005). Accordingly, in this study, three values of micromotion displacements (i.e., 1, 10, and 20 μm) were simulated on the stiff silicon-based Michigan probe. The model was extended to compare the strain values among four different probe materials: silicon, PVAc-NC, polyimide, and hypothetical probes for 1 μm displacement. In addition, a PVAc-NC probe with a thickness reduced to 25 μm, equal to the silicon probe thickness, was also considered under 1 μm displacement. A summary of the cases considered are presented in Table 1. From herein, silicon-based implants will be referred to as stiff probes while a PVAc-NC implant will be referred to as a compliant probe.

**TABLE 1:** The material properties, dimensions, and boundary conditions for each modeled probe and simulation case.

Case number	Implant material	Probe dimensions (μm)	Elastic modulus (MPa)	Poisson ratio	Applied displacement in *X*-axis direction (μm)
Case 1^a^	Silicon	1125 × 125 × 25	2×10^5^	0.278	1
Case 2^a^	Silicon	1125 × 125 × 25	2×10^5^	0.278	10
Case 3^a^	Silicon	1125 × 125 × 25	2×10^5^	0.278	20
Case 4^a^	Polyimide	1125 × 125 × 25	2.7×10^3^	0.33	1
Case 5^b^	Hypothetical soft	1125 × 125 × 25	6×10^−3^	0.33	1
Case 6^b^	PVAc-NC	1125 × 125 × 63	12.7	0.3	1
Case 7^b^	PVAc-NC	1125 × 125 × 25	12.7	0.3	1
Case 8^b^	PVAc-NC	1125 × 125 × 25	12.7	0.3	20

*^a^Probe dimensions and material properties are taken from Subbaroyan et al. (2005).*

*^b^Probe dimensions and modulus of elasticity are taken from Nguyen et al. (2014) and Poisson ratio from Polanco et al. (2016).*

### Geometry Modeling

**FIGURE 2:**
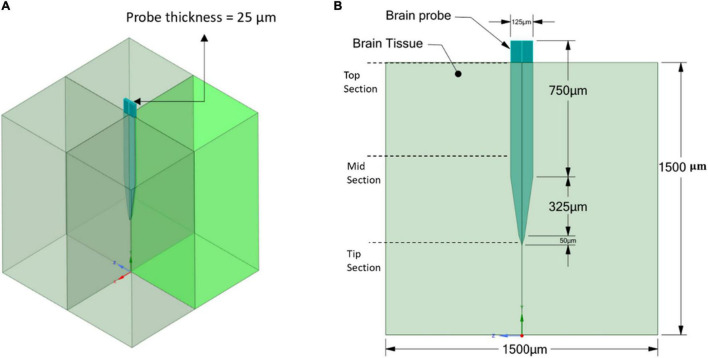
Stiff probe and brain tissue geometrical model and dimensions. **(A)** 3D view; **(B)** 2D front view.

**FIGURE 3:**
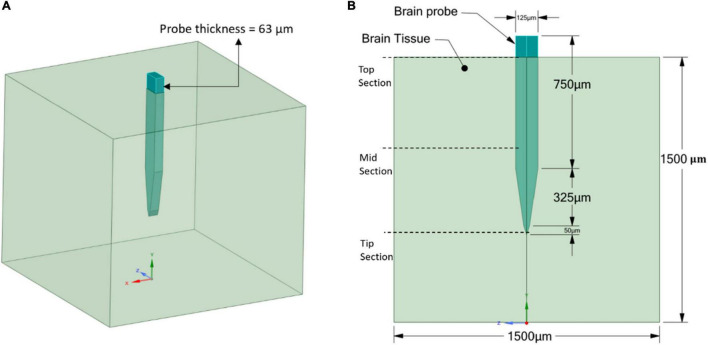
Compliant probe and brain tissue geometrical model and dimensions. **(A)** 3D view; **(B)** 2D front view.

The geometry of the model consists of two parts: the brain tissue modeled as a 3D rectangular shape and a Michigan-type electrode, based on the typical design used for silicon microelectrode arrays (Subbaroyan et al., 2005), of two different thicknesses. The probe geometry with a thickness of 25 μm (Figure 2) was used to represent the stiff, Polyimide and hypothetical probes, while the probe geometry with a thickness of 63 μm (Figure 3) was used to represent the compliant probe since, as reported in Nguyen et al. (2014), the manufacturing process limits the thickness of compliant probes to less than 63 μm. For the brain tissue, the width and length were taken as 1,500 × 1,500 μm, which are much larger than the effective recording distance from the probe surface and the kill zone of a single microelectrode of 140 and 60 μm, respectively (Subbaroyan et al., 2005).

### Material Properties

The brain tissue was approximated as a linear elastic and isotropic material, as described in Subbaroyan et al. (2005), Nguyen et al. (2014), and Mahajan et al. (2020). That is because the implants were assumed to be placed in the brain tissue of the cerebral cortex, which is mainly composed of gray matter and behaves isotropically (Prange and Margulies, 2002). Additionally, for the order of strain magnitudes predicted in this study, the accuracy of a non-linear model compared to linear is found to be within 1.5%, according to Taylor and Miller (2004). Thus, the brain tissue elastic modulus was set at 6,000 KPa with a Poisson’s ratio of 0.45 (Subbaroyan et al., 2005). The material property specifications of the different modeled probes were taken from the literature (Subbaroyan et al., 2005; Nguyen et al., 2014) as follows: the stiff silicon material was defined with an elastic modulus of 200 GPa and Poisson’s ratio of 0.278; the Polyimide material with an elastic modulus of 2.7 GPa, much lower than that of silicon, and a Poisson’s ratio of 0.33; the hypothetical material with an elastic modulus of 6,000 KPa, equal to that of the brain tissue, and a Poisson’s ratio of 0.33; and lastly, for the compliant probe the material was considered to be made of tunicate cellulose nanocrystal (NC) with a PVAc coating dipped in dimethylformamide. This material structure allowed a mechanically adaptive implant with an elastic modulus of 5.2 GPa pre-insertion and 12.7 MPa post-insertion, with Poisson’s ratio of 0.38 and 0.3, respectively (Capadona et al., 2008; Nguyen et al., 2014). In the simulation, the post-insertion values were used for the compliant probe. All material properties for the different probes are summarized in Table 1.

### Boundary and Loading Conditions

Brain micromotions can lead to tethering forces acting on the implant when the implant’s platform is grounded into the cranium of the brain. For instance, the rotational acceleration of the head could result in the probe being displaced parallel or perpendicular to its axis. To model this, the general brain movement can be restricted and a fixed boundary condition at bottom of the tissue is usually applied to prevent large-scale global displacements and allow the local displacement around the implant [more detailed information can be found in Subbaroyan et al. (2005)]. The adhesion properties between the microelectrode and brain tissue were assumed to be of good adhesion and the contact type was specified as bonded (Subbaroyan et al., 2005). The effect of micromotion can be translated into a displacement of the microelectrode, which could range from 1 to 20 μm at the electrode surface (Polanco et al., 2016).

**FIGURE 4:**
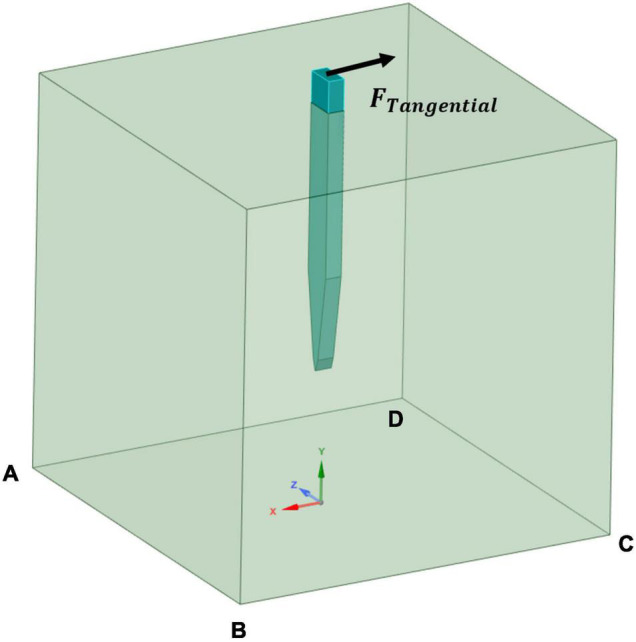
Simulation cases boundary conditions. A tangential load was applied at the probe’s upper surface and fixed supports defined at the edges of the bottom surface of the tissue model (AB, BC, CD, and DA).

In this study, the focus was on the tangential tethering force, and it is represented as a displacement load applied perpendicularly to the probe axis at the center of the top surface of the probe, while the edges of the base of the brain tissue were fixed. Figure 4 clarifies the location of the boundary load and the fixed supports at the edges of the bottom of the tissue domain. Different loading conditions of 1 μm, 10 μm, and 20 μm were applied on the stiff probe to determine whether the discrepancy in strain distribution prediction in the literature, between the model of Subbaroyan et al. (2005) and Nguyen et al. (2014), originates from the different loading conditions that was applied on a silicon-based probe. While for the rest of the different probes, a 1 μm displacement was considered. Table 1 summarizes the different displacements applied for different cases.

### Finite Element Analysis

A three-dimensional finite element model was used to simulate the probe-brain tissue interface and evaluate the strains formed in the tissue areas surrounding the probe as a function of different material properties and two probe sizes (Refer to Figures 2, 3). All of the simulations were performed under static conditions and using ANSYS Mechanical Biblography: Ansys^®^ Academic Research Mechanical, Release 18.1. The Von Mises strain output from the model was used for comparison between simulations.

### Domain Meshing

**FIGURE 5:**
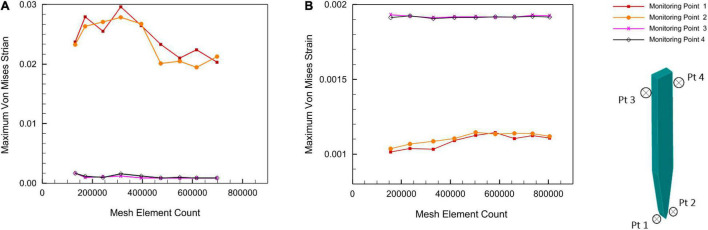
Mesh sensitivity analysis at a displacement of 1 μm. **(A)** Stiff silicon probe; **(B)** compliant PVAc-NC probe.

The full model for the brain probe–tissue was discretized with edge division seeding along the interfaces. A mesh sensitivity analysis on the stiff and compliant probe geometries was conducted. The maximum strain values at 4 different test points (i.e., two points close to the top probe–tissue interface and two points close to the probe tip–tissue interface) were plotted against increasing element density for a displacement of 1 μm (Figure 5). The results of the analysis showed that the variation of maximum strain as a function of mesh density, in the four monitoring points, was minimized above 400,000 elements.

**FIGURE 6:**
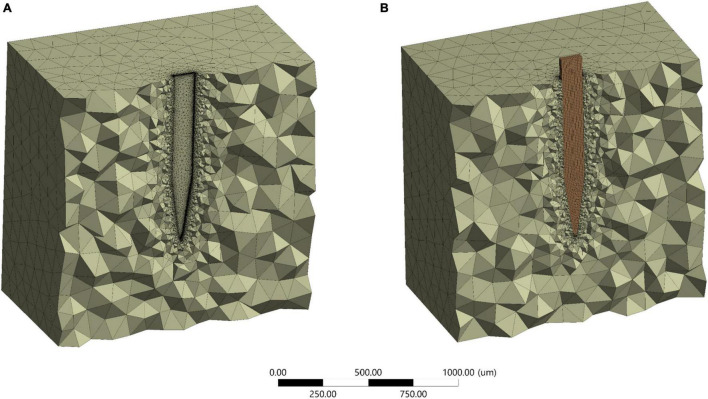
Final mesh half-section view. **(A)** Without probe; **(B)** with a probe.

Around 5% difference in the maximum Von Mises strain between 472,937 and the maximum number of elements of 700,000 for stiff probe, and 2% difference in maximum Von Mises strain between 416,145 and a maximum number of elements of 800,000 for the compliant probe. The maximum strain field at monitoring points 3 and 4, which are at the top probe-tissue interface in both stiff and compliant cases remained constant with the increase of elements number. Thus, for the stiff probe and compliant probe models, a total of 472,937 and 448,787 tetrahedron elements were used to mesh the domain geometry, respectively. The skewness and orthogonality for both cases were kept within the recommended range Ansys Academic Research Mechanical Biblography: Ansys Academic Research Mechanical, Release 18.1, User Guide, ANSYS, Inc. The final 3D meshed domain is shown in Figure 6 with and without a probe.

## Simulation Results

### Location of the Highest Strain for the Stiff Probe

**FIGURE 7:**
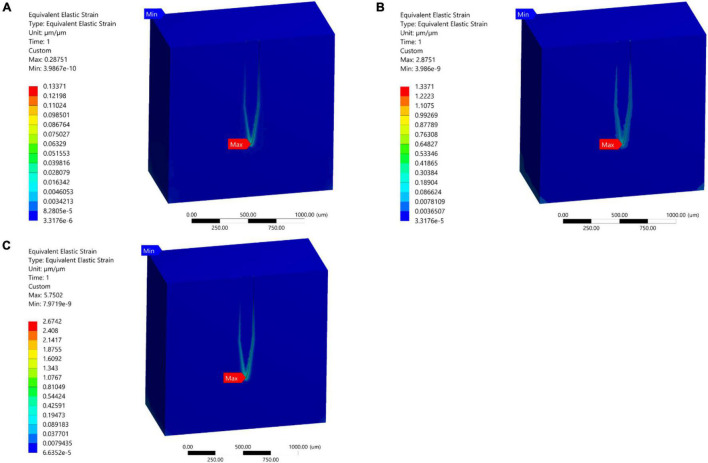
Mid-section views of the strain distribution for the stiff Michigan probe. **(A)** 1 μm – Case 1; **(B)** 10 μm – Case 2; **(C)** 20 μm – Case 3. The strain is concentrated at the tip of the probe and along the contact surfaces for the different loading conditions.

In the present study, 1, 10, and 20 μm displacements were applied on the top surface of the stiff probe. In all of the three cases, the model predicted that the highest tissue strain was always near the bottom tip area of the probe (Figures 7A–C). The results showed an increase in the strain distribution with the increase in displacement where the maximum prediction of elastic strain was 0.287, 2.8751, and 5.7502 for 1, 10, and 20 μm displacements, respectively.

### Stiff and Compliant Probe Comparison

**FIGURE 8:**
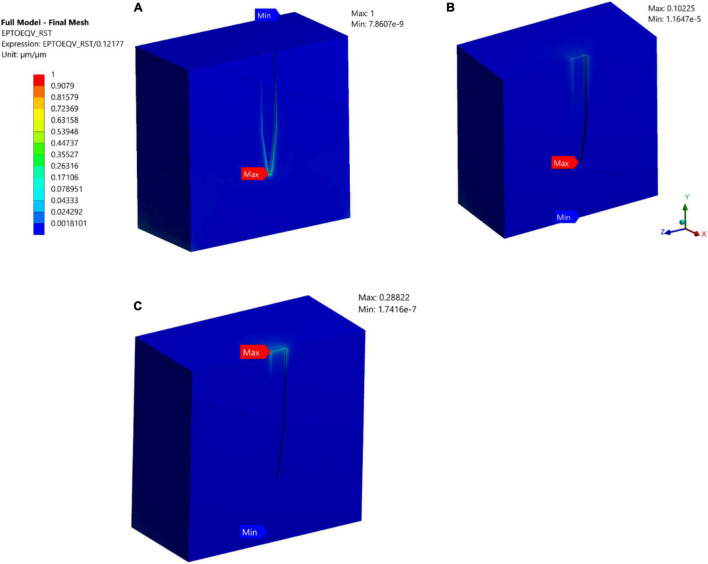
Normalized strain distribution. **(A)** Stiff probe with thickness = 25 μm – Case 1 in Table 1; **(B)** compliant probe with thickness = 63 μm – Case 6 in Table 1; **(C)** compliant probe with thickness = 25 μm – Case 7 in Table 1. Strain distributions are normalized to the maximum induced strain surrounding the stiff probe of Case 1 in Table 1.

A comparative analysis between a stiff probe and a compliant probe with two different thicknesses was undertaken to measure and quantify the effect of stiffness and compliant probe thickness on the strain fields. Stiff and compliant probe strain distributions were acquired at a 1 μm displacement for three cases (i.e., Case 1, 6, and 7) and the equivalent strain field distribution was normalized against the equivalent strain in the stiff case, which has a value of 0.1217 (i.e., the stiff probe strain outcomes act as a baseline case). The normalized maximum strain decreased drastically to 10.225% and 28% for the compliant probe with 63 and 25 μm, respectively, as illustrated in Figures 8B,C. Furthermore, the location of the maximum tissue strain was maintained at the surrounding tissue area of the tip for the 63 μm thick compliant probe, while it shifted to the top of the probe surrounding tissue area for the 25 μm compliant probe. Additionally, the strain distribution in the two cases of the compliant probe showed a higher concentration at the top of the probe–tissue interface in comparison to the stiff probe which has most of the strain concentrated on the tip of the probe–tissue interface.

### Strain Distribution

**FIGURE 9:**
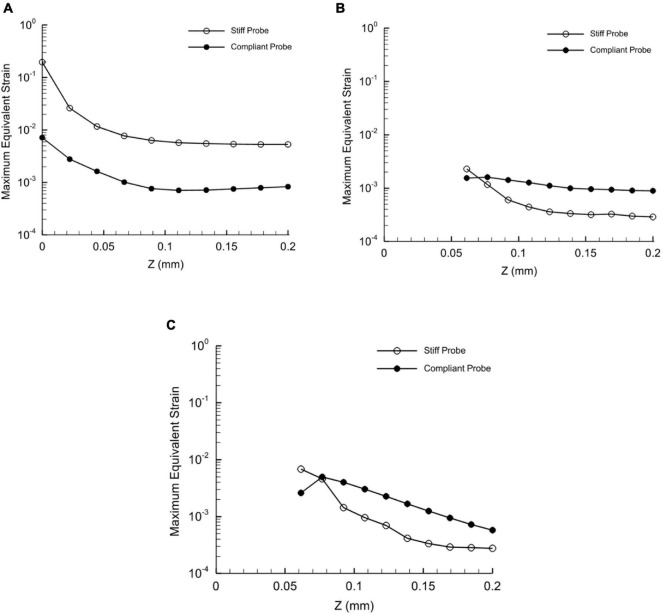
Distribution of the maximum equivalent strain of stiff and compliant probes with respect to distance in *Z*-axis direction at three different heights. **(A)** Tip of the probe; **(B)** mid of the probe; **(C)** top of the probe. The displacement applied on the two probes is 20 μm – Case 3 and 8 in Table 1. Since the thickness of the probe differs with height, the predictions in the plot **(B,C)** start at 62.5 μm from the probe axial axis. Refer to Figures 2, 3 for the height locations. Note that the *Y*-axis values are set to log scale.

To demonstrate the effect of compliant material properties on the induced strain distribution, charts of equivalent strain fields for stiff and compliant implants with 63 μm thickness at three locations: top, mid-level, and tip section (Refer to Figures 3, 4 for the section locations) of the probe were plotted as a function of the perpendicular distance to the thickness surface of the probes. The displacement applied to the probe surface was 20 μm. The charts show an exponential strain field decaying away from both implants. Moreover, the probe-induced strain spanned up to 200 μm from the probe surface. Next to the probe surface, the highest maximum strain for the stiff and compliant were 0.19 and 0.007, respectively, and they were located at the tip surrounding section. Interestingly, at the top and mid-sections, 65% higher strain magnitudes were predicted away from the probe surface for the compliant implant compared to that for the stiff implant (Figure 9).

### Polyimide and Hypothetical Probe Comparison

**FIGURE 10:**
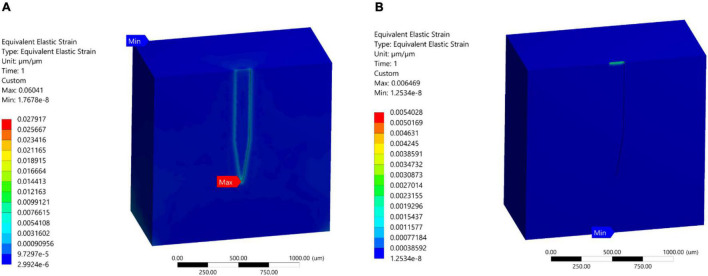
Strain field distribution at probe surroundings tissue region. **(A)** Polyimide (*E* = 2.7 GPa); **(B)** hypothetical soft material (*E* = 6 KPa).

Simulation of case 4 indicated that the use of polyimide as probe material reduced the magnitudes of maximum strain fields by up to 81% in comparison with the stiff implant under 1 μm displacement. Additionally, the strain distribution with the polyamide probe became more uniform along the tissue-probe interface with the maximum strain predicted at the probe tip surrounding region (Figure 10A). On the other hand, the simulation of case 5 showed that for the hypothetical material the maximum strain was predicted at the top surface of the tissue probe interface. As expected, when the mismatch in material properties between the probe and the tissue decreased, the magnitude of maximum strain was diminished and reached 0.0064 (Figure 10B).

## Discussion

### Brain Micromotion, Probe Displacement, and Tissue Strain

#### Displacement Does Not Affect the Location of Maximum Strain

The maximum strain induced in neural tissue is well accepted as one of the precursors to the brain’s immune response in the form of a glial sheath (Gilletti and Muthuswamy, 2006). In this work, we investigated the impact of micromotion of the brain on the magnitude and location of the stain induced in the tissue.

Cranial micromotion has been measured and well documented in the past (Gilletti and Muthuswamy, 2006; Helton et al., 2011). Micromotion is dominated by the animal respiratory and vascular cycle (Gilletti and Muthuswamy, 2006). A detailed literature search showed that a few groups have investigated the micromotion of neural probes, most notably Subbaroyan et al. (2005) and Nguyen et al. (2014). The simulation results of developed models in Subbaroyan et al. (2005) and Nguyen et al. (2014) differed in the location of the maximum strain field for probes under displacement. Work done by Nguyen et al. (2014) suggested that the strain is maximum at the top surface level where the displacement load is being applied. The work by Subbaroyan et al. (2005) found that the maximum strain is at the tip of the probe, deep in the brain tissue. The two studies differed in the amount of displacement simulated, which were 1 μm and 20 μm for Subbaroyan et al. (2005) and Nguyen et al. (2014), respectively. Therefore, our model investigated the hypothesis that the amount of displacement can account for the difference between the two predictions in terms of the location of the maximum strain. We selected a stiff implant for comparison since it was simulated in both previous models, and a 1, 10, and 20 μm displacement was applied at the surface of the implant to determine the maximum strain (Figures 7A–C). The finite element model results for all displacement load cases (1, 10, and 20 μm) indicated that for a stiff probe material (i.e., purely silicon), the maximum strain was found to be at the tip of the probe–tissue interface irrespective of the magnitude of the displacement. These results match several histological studies that support immune response to stiff silicon probes occurring at the tip of the electrode (Stice et al., 2007; Stice and Muthuswamy, 2009).

#### Strain Beyond 20 μm

As for the magnitude of the induced strain field, our simulations showed an increase in strain by one order of magnitude when increasing the displacement from 1 to 10 μm. On the other hand, for higher displacement over 10 μm the change in the strain was relatively smaller. In fact, when increasing the displacement from 10 to 20 μm, the model predicted only a 50% increase in the magnitude of the strain. The characterization of the impact of the full range of micromotion on strain helps us better understand the biomechanics of the implant-tissue interface throughout the life of the implant. Our data suggest that the strain from the large inward displacements of brain tissue between 10 and 60 μm, for example, immediately following the administration of anesthesia (Gilletti and Muthuswamy, 2006), does not cause large changes in strain beyond 25 μm.

On the other hand, our data highlight the large impact of micromotion on the magnitude of strain in the lowest range of 1–10 μm (almost an order of magnitude increase), which coincides with both cardiovascular (1–3 μm) and respiratory activity (6–10 μm). Also, it is worth mentioning that work done on quantifying brain micromotion in anesthetized animals showed that it ranged between 1 and 25 μm for various locations of implantation in the cortex (Gilletti and Muthuswamy, 2006).

### Impact of Compliant Probes on Strain Magnitude and Distribution

#### Polyimide and Hypothetical Probe

An example of polymers used as a backbone for neural interfaces is polyimide (PI), which is known for its superior thermal and chemical resistance, excellent electrical and thermal insulation of metallic conductors, biocompatibility, and high elasticity (Khraiche et al., 2017). That being said, PI still suffers from a mechanical mismatch with brain tissue due to its high elastic modulus (Bilston, 2011; Almasri et al., 2020). Our simulation results (Figure 10A) showed that neural probes made of PI will result in a large magnitude reduction of the strain fields (almost two orders of magnitude). In addition, the strain distribution became more uniform along all the interface edges (Case 4; Figure 10A). On the other hand, our simulations showed that the maximum strain occurs at the tip of the probe–tissue interface, which disagrees with the model results of Subbaroyan et al. (2005). As for simulations of the hypothetical probe, the results are not surprising as the softer material has an elastic modulus very close to that of brain tissue, which allows for a significant reduction in mechanical mismatch and negligible strain (Figure 10B).

#### Polyvinyl Acetate Nanocomposite Probe

As previously mentioned, while PI induces less strain compared to stiffer materials, it still suffers from a mechanical mismatch with brain tissue. With the potential advent of new and more compliant materials, we simulated softer and more compliant material probes. PVAc-NC gained attention due to its high stiffness prior to insertion (5.2 GPa), which allows tissue penetration, and then reduced stiffness following implantation to ∼12 MPa, bringing it much closer to the brain elastic modulus (6,000 KPa) compared to the other probe material. We compared a stiff microelectrode (Case 1; 200 MPa) and a compliant PVAc-NC based microelectrode [Case 6; 12.8 MPa; (Nguyen et al., 2014)] to measure and quantify the effect of the difference in material properties on the strain fields. Stiff and compliant electrodes were simulated at 1 μm displacements and found that strain was decreased by orders of magnitude for the compliant probe. The normalized maximum strain decreased to 10 percent for the compliant electrode, as illustrated in Figure 8 which agrees with the predictions in Nguyen et al. (2014). On the other hand, for the compliant implant (Case 6), the location of the maximum strain was still at the surrounding region of the probe tip-tissue interface (10.255%), but most of the strain was distributed around the top-tissue interface and with a very close value (i.e., approximately 10.1%) to the maximum strain at the tip -tissue interface. Additionally, we simulated another compliant implant with a thickness of 25 um, similar to the stiff implant thickness, to determine what would be the effect of fabricating a thinner compliant implant on strain magnitudes and distribution (i.e., Case 7 in Table 1). The model predicted higher maximum strain compared to the thicker implant, indicating that there is more bending of the probe at the displacement point of application. Furthermore, the prediction of the location of the maximum strain shifted to the top agreeing with the results of Nguyen et al. (2014) on compliant implants.

### Axial Strain Across Probe Body

Based on the axial strain distribution at the tip, mid and surface levels of the probe (i.e., perpendicular to the probe thickness) in Figure 9. The values of the strain at the tip decreased in the case of compliant in comparison to the stiff implant. Also, the strain dropped sharply below around 5 μm from the probe, which is in line with the size of the typical glial response to the neural probe of approximately 5–10 μm (Spencer et al., 2017). Our collected data in Figure 9 and coupled with published histological data suggest that the distribution of the implant surrounding strain can be potentially a determinant of the size of the glial sheath. On the other hand, at the top and mid sections, the compliant implant induced higher strain values than the stiff implant as the distance increased (Figure 9). Nevertheless, the strain values were very small compared to the tip values.

### New Designs, Challenges, and Future Direction

Current development in probe designs such as microwires, mesh electronics, and polymers, or the micromachining processes are a promising solution in reducing the effect of micromotions on the longevity of the probes (Khraiche et al., 2013b; Silva Gabriel et al., 2015; Szostak et al., 2017). Recently, a study on ultrafine microwires bundles interwoven into tabular braids showed a significantly less tissue immune response and more neural survivability than a 50 μm wire (Kim et al., 2019). They suggest that despite the material modulus mismatch between neural tissue and electrode build materials, a geometrical design that is more mechanical compliant and with small diameters, such as ultrafine wires, can have significant improvement in minimizing tissue inflammatory response (Kim et al., 2019). Another interesting design is the mesh electronics–tissue interface, which exhibits almost no chronic immune response up to at least a year post-injection due to their effective bending stiffness that is comparable to that of the neural tissue (Hong and Lieber, 2019). However, such designs still face challenges during the insertion as well as being limited by a smaller number of recording sites (Hong and Lieber, 2019). Alternatively, new flexible and wearable sensors consisting of liquid metals are being investigated (Ren et al., 2020). They are characterized with having excellent flexibility, conductivity, stretchability, and precision. Such innovative probes are the focus of our future simulations to expand our understanding of their designs and possible improvements.

## Conclusion

Penetrating 3D structures remain a viable approach for recording from the brain for both extracellular and, potentially, intracellular recordings (Spencer et al., 2017; Khraiche and El Hassan, 2020). Modeling of the complex tissue/implant biomechanical interaction can help drive developments in the design and implantation of microelectrodes in the cortex and could aid in further mitigating the chronic neuroinflammatory response. In addition, the mechanical strain has the potential to not only induce immune response but also neural modulation. Understanding induced strain conditions can help extend biomechanical neural modulation models to help better understand its effect on neural excitability (Tyler, 2012).

In our work, we simulated the complete range of cortical motion and its impact on the strain, which had not been undertaken previously in the literature. This resolves some of the discrepancies in published data and provides an understanding of the strains induced in the tissue due to the implant for the various micromotions. We showed that for a stiff implant, the strain magnitude is dependent on the magnitude of the displacement, however, the displacement magnitude has no impact on the location of maximum strain. Additionally, we examined the effect of several materials of implants on the magnitude, location, and distribution of strain. Finally, our data also indicate the potential for using the distribution of the implant’s surrounding strain as a determinant of the size of the glial sheath.

## Data Availability Statement

The raw data supporting the conclusions of this article will be made available by the authors, without undue reservation.

## Author Contributions

AA conceived the presented idea. AA and MK developed the theory and the methodology section. AA performed the numerical simulations. MK and JA verified the methods and supervised the findings of this work. All authors discussed the results and contributed to the final manuscript. All authors listed made an intellectual contribution to the work.

## Conflict of Interest

The authors declare that the research was conducted in the absence of any commercial or financial relationships that could be construed as a potential conflict of interest.

## Publisher’s Note

All claims expressed in this article are solely those of the authors and do not necessarily represent those of their affiliated organizations, or those of the publisher, the editors and the reviewers. Any product that may be evaluated in this article, or claim that may be made by its manufacturer, is not guaranteed or endorsed by the publisher.
